# Supplementary material for: Blockade of the M1 muscarinic acetylcholine receptors impairs eyeblink serial feature-positive discrimination learning in mice
Source: PLoS One. 2020 Aug 13;15(8):e0237451. doi: 10.1371/journal.pone.0237451 (PMC7425847; doi:10.1371/journal.pone.0237451)
Supplement: Attachment — Submitted filename: Response to Reviewers.doc [file pone.0237451.s002.doc]

**Response to Reviewers**

We thank the reviewers for reading the manuscript carefully and providing productive comment. We have subsequently revised our manuscript titled "Blockade of the M1 Muscarinic Acetylcholine Receptors Impairs Eyeblink Serial Feature-Positive Discrimination Learning in Mice.”

The comments made by each reviewer are first copied below (underlined) and followed by our responses. Each line number indicated with corresponding page number in our reply refers to that in the ‘Revised Manuscript with Track Changes’, in which the lines are numbered throughout the manuscript.

Reviewers' comments:

Reviewer's Responses to Questions

**Comments to the Author**

1. If the authors have adequately addressed your comments raised in a previous round of review and you feel that this manuscript is now acceptable for publication, you may indicate that here to bypass the “Comments to the Author” section, enter your conflict of interest statement in the “Confidential to Editor” section, and submit your "Accept" recommendation.

Reviewer#1: All comments have been addressed

Reviewer #2: All comments have been addressed

**Response:** We thank for the careful reading of our manuscript.

2. Is the manuscript technically sound, and do the data support the conclusions?

The manuscript must describe a technically sound piece of scientific research with data that supports the conclusions. Experiments must have been conducted rigorously, with appropriate controls, replication, and sample sizes. The conclusions must be drawn appropriately based on the data presented.

Reviewer#1: Yes

Reviewer #2: Yes

**Response:** We thank for the cautious reading of our manuscript.

3. Has the statistical analysis been performed appropriately and rigorously?

Reviewer#1: Yes

Reviewer #2: Yes

**Response:** We again thank for the careful reading of our manuscript.

4. Have the authors made all data underlying the findings in their manuscript fully available?


Reviewer#1: Yes

Reviewer #2: Yes

**Response:** We acknowledge for the careful reading of our manuscript.

5. Is the manuscript presented in an intelligible fashion and written in standard English?

PLOS ONE does not copyedit accepted manuscripts, so the language in submitted articles must be clear, correct, and unambiguous. Any typographical or grammatical errors should be corrected at revision, so please note any specific errors here.

Reviewer#1: Yes

Reviewer #2: Yes

**Response:** We thank for the careful reading of our manuscript.

-------------------------------------------------------------------------------------------------

5.Review Comments to the Author
***Reviewer #1:***

The authors have been sufficiently receptive and responsive to specific comments from the initial review. Their paper is technically sound and the experiments are well thought out. However, reservations regarding the impact of these studies in isolation persist. While the authors correctly point out that 40-50% of muscarinic receptors are M1, there are 50-60% of muscarinic receptors that are not M1. Also, while M2 only plays a minor role in this type of memory, it still serves some role and could produce similar effects.

Further, while this is a hippocampal dependent task, that necessarily means that this task is also dependent on the integrity of afferent inputs to hippocampus, particularly from other basal forebrain structures where M1 receptors are expressed. While the authors have appropriately tempered the scope of their findings, systemic administration of a selective agonist absent dissociation from other muscarinc receptors, and without any local specificity does not appreciably build on current literature.

**Response:** We thank for the careful reading. In response to this comment, we have made some changes in the different parts of manuscript.

We have added some lines (lines 81-93 in page 4, lines 96-98 in page 5, lines 106-107 in page 5) and deleted one line in the introduction part (see revised manuscript with track changes indicated by strike-through; lines 89-90 in page 4). We have added the corresponding references lines 560-572 in pages 24-25 and lines 585-587 in page 25) as below

In the hippocampus-dependent trace eyeblink conditioning it was shown that selective activation of the M1 receptors improved the memory in aged rabbits by enhancing the excitability of hippocampal pyramidal neurons [38]. Consistent with this, an electrophysiological study using mAChRs knock-out mice revealed that the M1 but not the M3 receptors are involved in the cholinergic enhancement of hippocampal long-term potentiation [39]. Immunohistochemical study also showed a selective increase in immunoreactivity of PKCγ isoform after trace eyeblink conditioning, suggesting the involvement of signaling pathways of M1 receptors [40]. In contrast to the abundance of M1 receptors in the pyramidal neurons [41] which are highly engaged in learning the hippocampus-dependent eyeblink [38], the M2 receptors are present only in the nonpyramidal neuron in the cortex and hippocampus [27]. In addition, they are densely expressed on GABAergic interneurons [42].

Consistent with this, a significant correlation was found between the performance in spatial learning and the M1 receptor binding, but not the M2, in the hippocampus of aged monkeys [47].

Therefore, we focused on the M1 receptors as the first candidate that plays an important role in the serial feature-positive discrimination task of eyeblink conditioning, although the other subtypes of mAChRs that constitute 50‒60% of the hippocampal mAChRs might serve some roles in this learning as well.

Also, we have added some lines (lines 373-375 in page 16), modified the sentence by using “particularly the basal forebrain, which” (line 386 in page 17) in the discussion part and “systemic administration of” (line 443 in page 19) in the conclusion part as below

In addition, there might be some contribution of extrahippocampal structures, such as the basal forebrain, which express the M1 mAChRs [41] and have close interaction with the hippocampus [5].

**References**

5. Mckinney MI, Coyle JT, Hedreen JC. Topographic analysis of the innervation of the rat neocortex and hippocampus by the basal forebrain cholinergic system. Journal of Comparative Neurology. 1983; 217(1): 103‒21.
27. Levey AI, Edmunds SM, Koliatsos V, Wiley RG, Heilman CJ. Expression of m1-m4 muscarinic acetylcholine receptor proteins in rat hippocampus and regulation by cholinergic innervation. Journal of Neuroscience. 1995; 15(5): 4077‒92.
38. Weiss C, Kronforst‐Collins MA, Disterhoft JF. Activity of hippocampal pyramidal neurons during trace eyeblink conditioning. Hippocampus. 1996; 6(2): 192-209.
39. Shinoe T, Matsui M, Taketo MM, Manabe T. Modulation of synaptic plasticity by physiological activation of M1 muscarinic acetylcholine receptors in the mouse hippocampus. Journal of Neuroscience. 2005; 25(48): 11194-200.
40. Van der Zee EA, Kronforst‐Collins MA, Maizels ET, Hunzicker‐Dunn M, Disterhoft JF. γisoform‐selective changes in PKC immunoreactivity after trace eyeblink conditioning in the rabbit hippocampus. Hippocampus. 1997; 7(3): 271-85.
41. Levey AI. Muscarinic acetylcholine receptor expression in memory circuits: implications for treatment of Alzheimer disease. Proceedings of the National Academy of Sciences. 1996; 93(24): 13541‒6.
42. Ballinger EC, Ananth M, Talmage DA, Role LW. Basal forebrain cholinergic circuits and signaling in cognition and cognitive decline. Neuron. 2016; 91(6): 1199-218.
47. Haley GE, Kroenke C, Schwartz D, Kohama SG, Urbanski HF, Raber J. Hippocampal M1 receptor function associated with spatial learning and memory in aged female rhesus macaques. Age. 2011; 33(3): 309-20.

Beside these modification, we have made some changes by adding “the eyeblink” in place of “an eyeblink” (line 44 in page 2), “the” and “receptors” (line 96 page 5) and “eyeblink’ (line 365 in page 16)

Reviewer #2: (No Response)

**Response:** We acknowledge for the careful reading.
